# Plasma p-tau217 measured by the Elecsys automated immunoassay: Prospective validation in a heterogeneous memory clinic cohort

**DOI:** 10.1177/13872877261459067

**Published:** 2026-06-16

**Authors:** Emilio Franco-Macías, José Ángel Noval-Padillo, Rocío Hervás-Navidad, Beatriz Espejo-Martínez, Cristina Serrano-Gutiérrez, Silvia Rodrigo-Herrero, Carlota Méndez-Barrio, Gonzalo Mendoza-Vázquez, Ismael Carrera-Muñoz, Bartolomé Marín-Romero, Francisco Garzón-Maldonado, Lina Carazo-Barrios, Lucía Rodríguez-Jiménez, Ángel Martínez-Nogueras, Eduardo Agüera-Morales, Cristina Conde-Gavilán, Ana María Jover, Carmen García-Navarro, Mar Iglesias-Espinosa, David José Romera-Morales, Eva Cuartero-Rodríguez, Ernesto García-Roldán, Melisa González-Acosta, Ángela Almodóvar-Sierra, Marta Marín-Cabañas, María Bernal Sánchez-Arjona, María Isabel García-Sánchez, José Enrique Arriola-Infante, Carlos Arrabal-Gómez, Begoña Oliver-Martos, Pedro Jesús Serrano-Castro, Daniel Fatela-Cantillo

**Affiliations:** 1 Memory Unit, Department of Neurology, 16885 Virgen del Rocío University Hospital, Seville, Spain; 2 Biomedicine Institute of Seville IBiS, 16885 University Hospital Virgen del Rocio, CSIC, University of Seville, Seville, Spain; 3 Andalusia–NEURO-RECA/Roche Alliance in Precision Neurological Medicine, Spain; 4 Clinical Biochemistry Department, 16885 Virgen del Rocío University Hospital, Seville, Spain; 5 Memory Unit, Department of Neurology, 16581 San Cecilio University Hospital, Granada, Spain; 6 Memory Unit, Department of Neurology, 16839 Juan Ramón Jiménez University Hospital, Huelva, Spain; 7 Memory Unit, Department of Neurology, 16504 Virgen de las Nieves University Hospital, Granada, Spain; 8 Memory Unit, Department of Neurology, 16867 Virgen de la Victoria University Hospital, Málaga, Spain; 9 Memory Unit, Department of Neurology, 16842 Jaén University Hospital, Jaén, Spain; 10 Memory Unit, Department of Neurology, 16501 Reina Sofía University Hospital, Córdoba, Spain; 11 Memory Unit, Department of Neurology, 16815 Torrécardenas University Hospital, Almería, Spain; 12 Memory Unit, Department of Neurology, 16583 Virgen de Valme University Hospital, Seville, Spain; 13 Methodological and Statistical Support Unit, Fundación para la Gestión de la Investigación en Salud de Sevilla (FISEVI), Seville, Spain; 14 Biobank of the Andalusian Public Health System, Seville, Spain; 15 Department of Neurology, Regional University Hospital, Málaga, Spain; 16 Biomedical Research Institute and the Nanomedicine Platform—IBIMA BIONAND Platform, Málaga, Spain; 17 Department of Animal Physiology, Cell Biology and Genetics, 16330 University of Málaga, Málaga, Spain

**Keywords:** Alzheimer's disease, biomarkers, blood biomarkers, laboratory techniques/procedures

## Abstract

**Background:**

Plasma phosphorylated tau at threonine 217 (p-tau217) has emerged as a leading blood-based biomarker for the diagnosis of Alzheimer's disease (AD) and can be measured using fully automated, random-access platforms. The Elecsys plasma p-tau217 assay requires further validation, particularly in heterogenous populations seen in memory clinics.

**Objective:**

To validate plasma p-tau217 in comparison with p-tau181 and to evaluate its association with other soluble core 1 AD biomarkers, as well as markers of neurodegeneration and neuroinflammation.

**Methods:**

Biobank data from two prospective blood-based biomarkers validation studies were analyzed. Cerebrospinal fluid (CSF) p-tau181/Aβ_42_ ratio served as the reference standard for AD diagnosis. The diagnostic performance of plasma p-tau217 and p-tau181 was compared. In patients with AD, p-tau217 was further evaluated for its association with CSF (Aβ_42_/Aβ_40_ ratio, p-tau181, t-tau) and plasma [APOE ε4 protein (APOE ε4p), NfL (neurofilament light chain), GFAP (glial fibrillary acidic protein)] biomarkers.

**Results:**

Among 303 patients with mild cognitive impairment or mild dementia, plasma p-tau217 outperformed plasma p-tau181 (AUC 0.93 versus 0.87). By the two threshold diagnostic strategy, an upper cutoff (>0.312 pg/mL, specificity 95%) and a lower cutoff (<0.177 pg/mL, sensitivity 95%) were established, with 27% of cases falling into an indeterminate range. Plasma p-tau217 showed strong correlations with CSF Aβ_42_/Aβ_40_ and p-tau181/Aβ_42_ ratios, as well as with plasma GFAP, and only moderate correlations with CSF t-tau and p-tau181, and plasma NfL.

**Conclusions:**

Plasma p-tau217 measured using Elecsys demonstrates good diagnostic performance and strong associations with other soluble Core 1 AD and neuroinflammation biomarkers.

## Introduction

Early and accurate diagnosis has become a central priority in Alzheimer's disease (AD) with the recent introduction of disease-modifying therapies.^1,2^ Despite this progress, AD diagnosis remains challenging: up to 35% of patients in specialized clinics and 39% in primary care may be misclassified when biomarker support is unavailable.^3^ Until recently, pathophysiological biomarkers were accessible only through cerebrospinal fluid (CSF) analysis or positron emission tomography (PET) imaging with amyloid and tau tracers.^4^ Although diagnostically robust, these methods are limited by high cost, restricted availability, and invasiveness, constraining their widespread clinical use.^5,6^

The emergence of blood-based biomarkers (BBMs) represents a major advance in AD diagnostics. BBMs correlate with postmortem AD pathology, reliably distinguish AD from other causes of cognitive impairment, and predict progression from normal cognition and mild cognitive impairment to AD dementia.^7^ Their scalability, affordability, and patient acceptability offer the opportunity to substantially improve access to timely and accurate diagnosis.^3^ These advances have been enabled by highly sensitive technologies capable of quantifying brain-derived molecules in blood at very low concentrations.^8,9^ Importantly, BBMs have now been implemented on fully automated, high-throughput platforms, facilitating reproducibility and integration into routine clinical laboratory workflows.^10–13^ Among these, Lumipulse G and Elecsys,^10–12^ already widely used for CSF AD biomarker measurements in clinical laboratories worldwide, are developing BBMs assays.

A particularly important development in BBMs research has been the focus on phosphorylated tau (p-tau) species, including the phosphorylated tau at threonines 217 (p-tau217), 181 (p-tau181), and 231 (p-tau231).^14^ These biomarkers have demonstrated high diagnostic accuracy in the differential diagnosis of cognitive decline and strong prognostic value for predicting progression to AD dementia.^15^ In early AD, plasma p-tau levels increased more prominently than amyloid-β (Aβ) markers, reflecting a downstream physiological response to Aβ plaque accumulation and linking amyloid pathology to early tau proteinopathy.^16,17^ Consequently, the most recent NIA–AA diagnostic criteria classify plasma p-tau biomarkers as Core 1 biomarkers, indicative of AD neuropathological change.^18^

Among fully automated platforms, Lumipulse G has demonstrated particularly robust performance, with consistent results across multiple cohorts using plasma p-tau217.^11^ This biomarker shows larger fold changes than p-tau181 and p-tau231 and achieves high diagnostic accuracy, with areas under the curve (AUCs) exceeding 90%.^7,8^ However, further prospective real-world clinical studies are needed to assess its impact on AD diagnosis and clinical management.^19^

Using the cobas platform, the Elecsys plasma p-tau181 immunoassay has received FDA recommendation for use in primary care settings, largely due to its high negative predictive value.^20^ More recently, a plasma p-tau217 immunoassay has been introduced as a prototype but requires further validation, particularly through studies in memory clinic cohorts, to establish clinically relevant diagnostic cutoffs.^12^

Research into de role of plasma p-tau217 as biomarker in patients with cognitive impairment is rapidly advancing. Although some studies have found elevation with advancing clinical stages, increase over time, and association with cognitive measures,^21^ prospective cohort studies that take core 2 biomarkers into account position p-tau217 as a core 1 biomarker of AD pathology.^22^

Our aim was to validate the Elecsys p-tau217 assay for the detection of AD in clinical settings, comparing its diagnostic utility with that of other plasma biomarkers, including p-tau181, and to evaluate its association with other soluble core 1 AD biomarkers, and also with biomarkers of neurodegeneration and neuroinflammation.

## Methods

### Design

We conducted a cross-sectional, retrospective observational study using biobank data collected from two prospective BBMs validation studies.

### Setting

Biobank CSF and blood samples were recruited from two research projects conducted simultaneously in Memory Units of public hospitals in Andalusia (southern Spain) between August 2023 and September 2025:

- *“The Andalusian Neuro-Reca Alzheimer Cohort”* (ANRAC)”. This cohort study was developed by the “*Andalusia Neuro-RECA – Roche Alliance in Precision Medical Neurology*”, that is a public–private collaboration advancing personalized neurology through biomarkers and modern diagnostic tools to improve patient care and quality of life, conducted by a multicenter research project entitled “*Personalized Medicine in Neurological Diseases Through the Application of Biomarkers to Improve the Diagnosis, Prognosis and Treatment of the Patient*” (PIP-0123-2022). Its “*Alzheimer's Disease and Other Dementias Program”* (S2300168) involves Memory Units from nine leading academic centers across Andalusia: Torrecárdenas University Hospital (Almería); Jaén University Hospital (Jaén); Virgen de las Nieves University Hospital and San Cecilio University Hospital (Granada); Reina Sofía University Hospital (Córdoba); Virgen de la Victoria University Hospital (Málaga); Juan Ramón Jiménez University Hospital (Huelva); and Virgen de Valme University Hospital and Virgen del Rocío University Hospital (Seville). This program established the ANRAC cohort, which prospectively recruited patients with mild cognitive impairment (MCI) in whom prodromal AD was suspected.
- “*Validation of the plasma amyloid ratio for the diagnosis of Alzheimer's disease*” (S2300347). This cross-sectional BBMs validation study was conducted at the Memory Unit of Virgen del Rocío University Hospital (Seville). The study prospectively recruited patients from routine clinical practice with a clinical diagnosis of mild cognitive impairment (MCI) or mild dementia with suspected AD.

Both projects shared the primary objective of validating BBMs for the diagnosis of AD and were therefore integrated into the present study, entitled “*Validation of plasma biomarkers for the diagnosis of Alzheimer's disease using automated laboratory methods”* (S2500012). This cross-sectional study analyzed Biobank data from the two preceding projects, including sociodemographic, clinical, and cognitive variables, as well as blood and CSF biomarker measurements. Participants enrolled in either project met the same selection criteria:

- Prospective evaluation at a Memory Unit.
- Diagnosis of mild cognitive impairment or mild dementia, based on clinical, cognitive, and neuroimaging assessments.
- Clinical suspicion of AD as the underlying cause, or the need to rule it out.
- No contraindications to lumbar puncture
- Provision of written informed consent for both lumbar puncture and the donation of CSF and plasma samples to the Biobank for research purposes, including biomarker validation studies

### CSF and plasma biomarkers

#### Preanalytical process

CSF samples were collected by lumbar puncture under fasting conditions in the early morning, following standardized international recommendations,^23^ and processed according to the Elecsys Gen immunoassay protocol.^24–27^ The initial 1–2 mL was reserved for routine analyses. Validation samples (5 mL) were collected into two polypropylene tubes (2.5 mL each, Sarstedt, Germany). Within 2 h of collection, samples were centrifuged at 2000 × g for 10 min at room temperature, then aliquoted into 500-µL polypropylene tubes and stored at −80 °C until analysis.

Blood samples were collected immediately after lumbar puncture following recommended preanalytical protocols for plasma biomarkers of AD amyloid pathology to ensure reproducibility.^28^ Samples were drawn into two EDTA-K2 tubes and centrifuged within 2 h at 2000 rpm for 10 min at 4 °C. Plasma was aliquoted (≥0.5 mL) into 3.6 mL and 2.5 mL polypropylene cryotubes (ThermoFisher, USA) and stored at −80 °C until analysis. According with other preanalytical protocols biomarkers can be measured in plasma from biobanks up to 13 years, with minimal impact from long-term storage or other pre-analytical factors.^26,27^

CSF and blood samples were initially stored at the nodes of the Andalusian Public Health System Biobank associated with the hospital areas participating in projects S2300168 (multicenter) and S2300347 (single-center, Virgen del Rocío University Hospital). All samples were subsequently shipped from their respective biobank nodes to the Clinical Biochemistry Department of Virgen del Rocío University Hospital, where the analytical procedures were performed. Samples were transported under appropriate conditions of temperature control and traceability, on dry ice, to ensure sample integrity.

#### Analytical process

Deeply frozen CSF samples were thawed at room temperature (18–25°C) and vortexed for 5 s before loading onto the cobas e801 analyzer. CSF Aβ_42_, Aβ_40_, total tau (t-tau), and phosphorylated tau (p-tau181) were measured by electrochemiluminescence immunoassay (ECLIA) using the cobas e801 platform. Frozen samples were thawed at room temperature and analyzed within 2 h in the original cryotubes, with all 303 samples measured using the same reagent lots. Second-generation Elecsys^®^ β-Amyloid (1–42) CSF II, Elecsys^®^ Phospho-Tau (181P) CSF, and Elecsys^®^ Total-Tau CSF kits (Roche Diagnostics International Ltd, Rotkreuz, Switzerland) were used on the cobas analyzer (Roche Diagnostics GmbH, Mannheim, Germany). The automated assay was performed according to the manufacturer's guidelines, with calibration completed prior to analysis. The analytical measuring ranges were 150–2500 pg/mL for Elecsys Gen II Aβ_42_, 8–120 pg/mL for Elecsys pTau181, and 80–1300 pg/mL for t-tau. CSF Aβ_40_ was measured using a prototype immunoassay with a lower limit of quantitation of 0.123 ng/mL. All samples were analyzed as singlets in accordance with the manufacturer's protocol, due to the high within-run precision of the Elecsys system.^28^

On the day of analysis, plasma samples were thawed at room temperature, mixed thoroughly, centrifuged at 2000 × g for 5 min, and directly measured on the cobas platform. BBMs—including p-tau217, APOE ε4 protein (APOE ε4p), neurofilament light chain (NfL), and glial fibrillary acidic protein (GFAP)—were measured using Elecsys^®^ plasma prototype immunoassays on the cobas e801 platform by ECLIA. In parallel, plasma p-tau181 was measured using a CE-marked kit with a measuring range of 0.300–10.0 pg/mL. The analytical measuring ranges for the Elecsys prototypes were 0.0791–2.66 pg/mL for p-tau217, 0.5–5000 pg/mL for NfL, and 0.00128–8.0 ng/mL for GFAP. The APOE ε4p result was binarized at 0.9 µg/ml, which has proven to accurately distinguish between *APOE* ε4 carriers and non-carriers.^29^

### Statistical analysis

Analyses were performed using SPSS version 31 and R (R Core Team, 2024). Quantitative variables were described using measures of central tendency and variability, while qualitative variables were summarized using absolute and relative frequencies. The normality assumption for quantitative variables was assessed using the Kolmogorov-Smirnov test. Comparisons of numerical variables between groups were performed using the nonparametric Mann–Whitney U test or the Kruskal–Wallis test, as the assumption of normality was not met. Associations between qualitative variables were analyzed using contingency tables and either the Chi-square test or non-asymptotic methods.

Spearman's rank correlation was used to assess associations between continuous variables.

The diagnostic performance of the different biomarkers was evaluated using ROC curves, and the area under the curve (AUC) was calculated. Several logistic multivariate models were also constructed with different variables to assess whether predictive performance could be improved. Variable selection was performed using the glmulti package, variables showing multicollinearity were removed, and model validation was carried out using the DHARMa package. Given the strong predictive performance of the biomarkers reported in previous studies, biomarkers with an AUC ≥ 0.85 were selected and compared with the multivariate models using DeLong's test, with Bonferroni correction applied for multiple comparisons.

To analyze the effect of age on the diagnostic accuracy of plasma p-tau217, the cohort was stratified using the median age, as this variable followed a non-normal distribution.

For plasma p-tau217 and p-tau181, a two-cutoff strategy was applied to define three zones. For the lower cutoff, a minimum sensitivity of 95% was required while maximizing the negative predictive value, and for the upper cutoff, a minimum specificity of 95% was required while maximizing the positive predictive value. These cutoffs were calculated robustly by generating 200 bootstrap samples, and out-of-sample performance was evaluated using 1000 bootstrap samples with the cutpointr package. We evaluate the overall accuracy, defined as the sum of true positives and true negatives divided by the total number of patients outside the intermediate range.

Finally, AD patients were stratified into three plasma p-tau217 strata using the upper cut-off established in the general cohort and the median plasma p-tau217 value among AD patients above that cut-off. Comparisons between groups were then performed using other CSF and plasma biomarkers.

A seed of 123 was set to ensure reproducibility of the results.

### Results

A total of 303 biobank records were included: 131 from study S2300168 and 172 from study S2300347. Patients in MCI stage (n = 209, 69%) outnumbered those in mild dementia stage (n = 94, 31%). According to the established cut-off for AD diagnosis (CSF P-tau181/Aβ42 ratio ≥ 0.024, based on Elecsys), 189 patients (62.4%) were diagnosed with AD, while 114 patients (37.6%) received a non-AD diagnosis (ratio < 0.024). Compared with the non-AD group, the AD group was older, included a higher proportion of women, and had higher educational attainment, mainly due to a larger proportion of patients with education beyond primary school (Table 1). Both groups were similar in the proportion of patients at the MCI stage and mild dementia stage (Table 1). Global cognition, by MMSE total score, was lower in the AD group.

**Table 1.** Sociodemographic and clinical characteristics of patients (total sample and by diagnostic group.

	Total sample (n = 303)	AD (n = 189, 62.4%)	Non-AD (n = 114, 37.6%)	p
Age M (IQR)	73 (67–77)	73 (69–77)	70 (64–75)	<0.001
Sex (female) n (%)	143 (47.2%)	102 (54%)	41 (36%)	<0.005
Education level n (%)				<0.005
<First grade	67 (22.1%)	42 (22.2%)	25 (21.9%)	
First grade	103 (34%)	51 (27%)	52 (45.6%)	
>First grade	133 (43.9%)	96 (50.8%)	37 (32.5%)	
Clinical stage n (%)				
MCI	209 (69%)	132 (69.8%)	77 (67.5%)	0.675
Mild dementia	94 (31%)	57 (30.2%)	37 (32.5%)	
MMSE M (IQR)	24 (22–26)	24 (22–26)	24 (22–28)	<0.05

Categorical variables are presented as frequency (percentage), and continuous variables as median (interquartile range). AD: Alzheimer's disease; Non-AD: non-Alzheimer's disease; M: median; IQR: interquartile range; MCI: mild cognitive impairment; MMSE: Mini-Mental State Examination.

**Figure 1.**
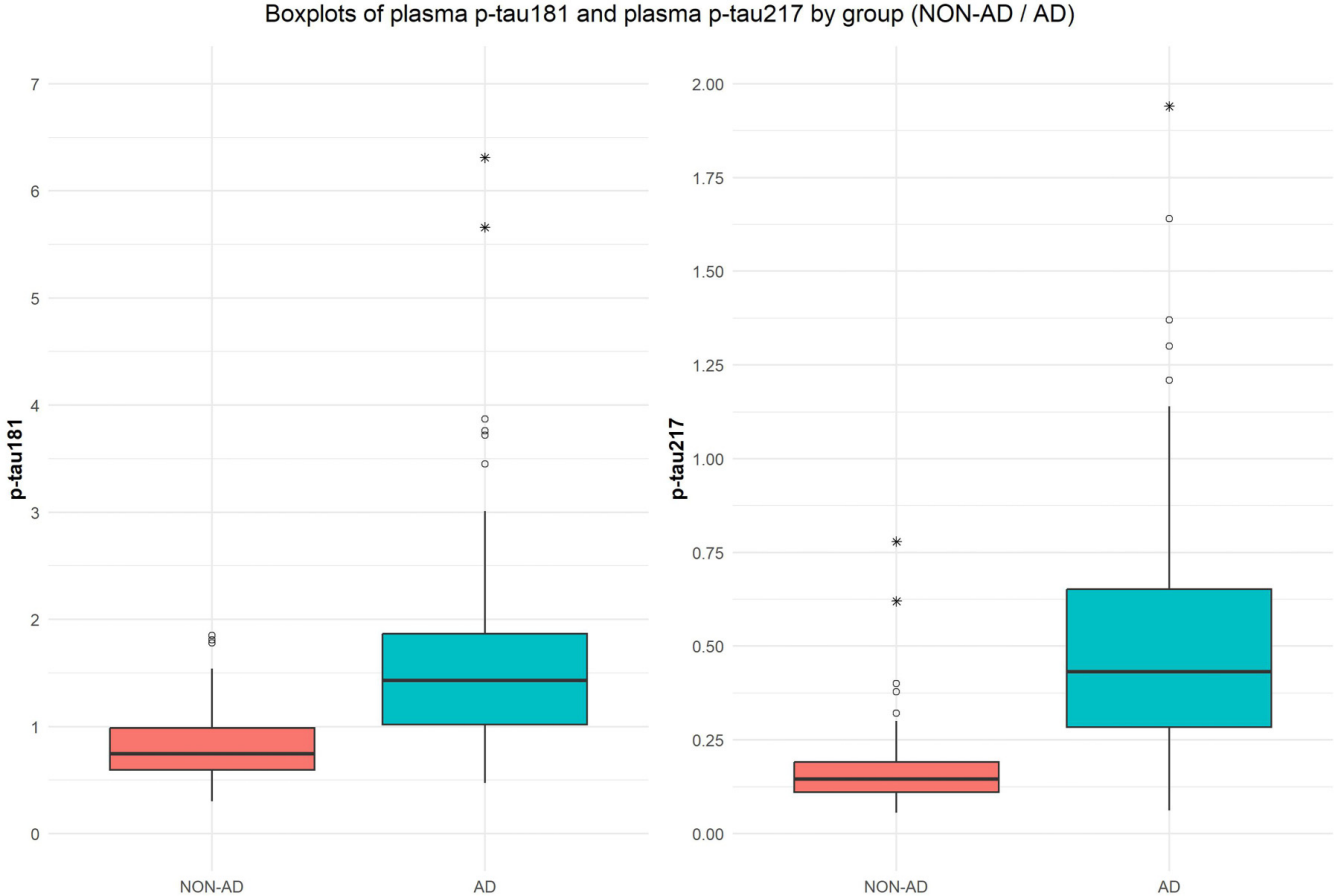
Boxplots comparing p-tau181 and p-tau217 between Alzheimer's disease and non-Alzheimer's disease groups.

Regarding CSF biomarkers, as expected, the AD group exhibited a lower Aβ_42_/Aβ_40_ ratio, higher t-tau and p-tau181 levels, and a higher p-tau181/Aβ_42_ ratio compared with the non-AD group (Table 2). In plasma, patients with AD showed significantly higher values of p-tau217, p-tau181, NfL, and GFAP (Table 2). Median plasma p-tau217 levels were more than threefold higher in the AD group than in the non-AD group [0.43 pg/mL (0.28–0.66) versus 0.15 pg/mL (0.11–0.19); *p* < 0.001]. In contrast, plasma p-tau181 concentrations differed by approximately twofold between groups [1.44 pg/mL (1.02–1.88) versus 0.75 pg/mL (0.59–0.99; *p* < 0.001)] (Table 2; Figure 1). Within the AD group, plasma p-tau217 and p-tau181 levels did not differ significantly across clinical stages. The proportion of *APOE* ε4 carriers was higher in the AD group (56.6% versus 12.3%).

**Table 2.** Levels of biomarkers in CSF and plasma (total sample and by group diagnostic).

		Total sample (n = 303)	AD (n = 189, 62.4%)	Non-AD (n = 114, 37.6%)	p
CSF	Ratio Aβ_42_/Aβ_40_	0.035 (0.026–0.058)	0.028 (0.024–0034)	0.063 (0.055–0.073)	<0.001
	t-tau (pg/ml)	230 (170–318)	272 (209–373)	177 (130–212)	<0.001
	p-tau181 (pg/ml)	22 (16–33)	29 (21–39)	16 (12–20)	<0.001
	Ratio p-tau181/Aβ_42_	0.035 (0.016–0.061)	0.053 (0.038–0.077)	0.014 (0.011–0.018)	<0.001
Plasma	APOE ε4p, n (%)	121 (33.9%)	107 (56.6%)	14 (12.3%)	<0.001
	p-tau217 (pg/ml)	0.28 (0.16–0.52)	0.43 (0.28–0.66)	0.15 (0.11–0.19)	<0.001
	p-tau181 (pg/ml)	1.09 (0.77–1.54)	1.44 (1.02–1.88)	0.75 (0.59–0.99)	<0.001
	NfL (pg/ml)	2.32 (1.71–3.23)	2.44 (2.00–3.28)	1.94 (1.37–2.79)	<0.001
	GFAP (ng/ml)	0.10 (0.07–0.15)	0.13 (0.09–0.17)	0.06 (0.05–0.09)	<0.001

Categorical variables are presented as frequency (percentage), and continuous variables as median (interquartile range). AD: Alzheimer's disease: Non-AD: non-Alzheimer's disease; M: median; IQR: interquartile range; CSF: cerebrospinal fluid; NfL: neurofilament light chain; GFAP: glial fibrillary acidic protein; APOE ε4p (a concentration ≥ 0.9 µg/ml indicates *APOE* ε4 carrier status).

**Figure 2.**
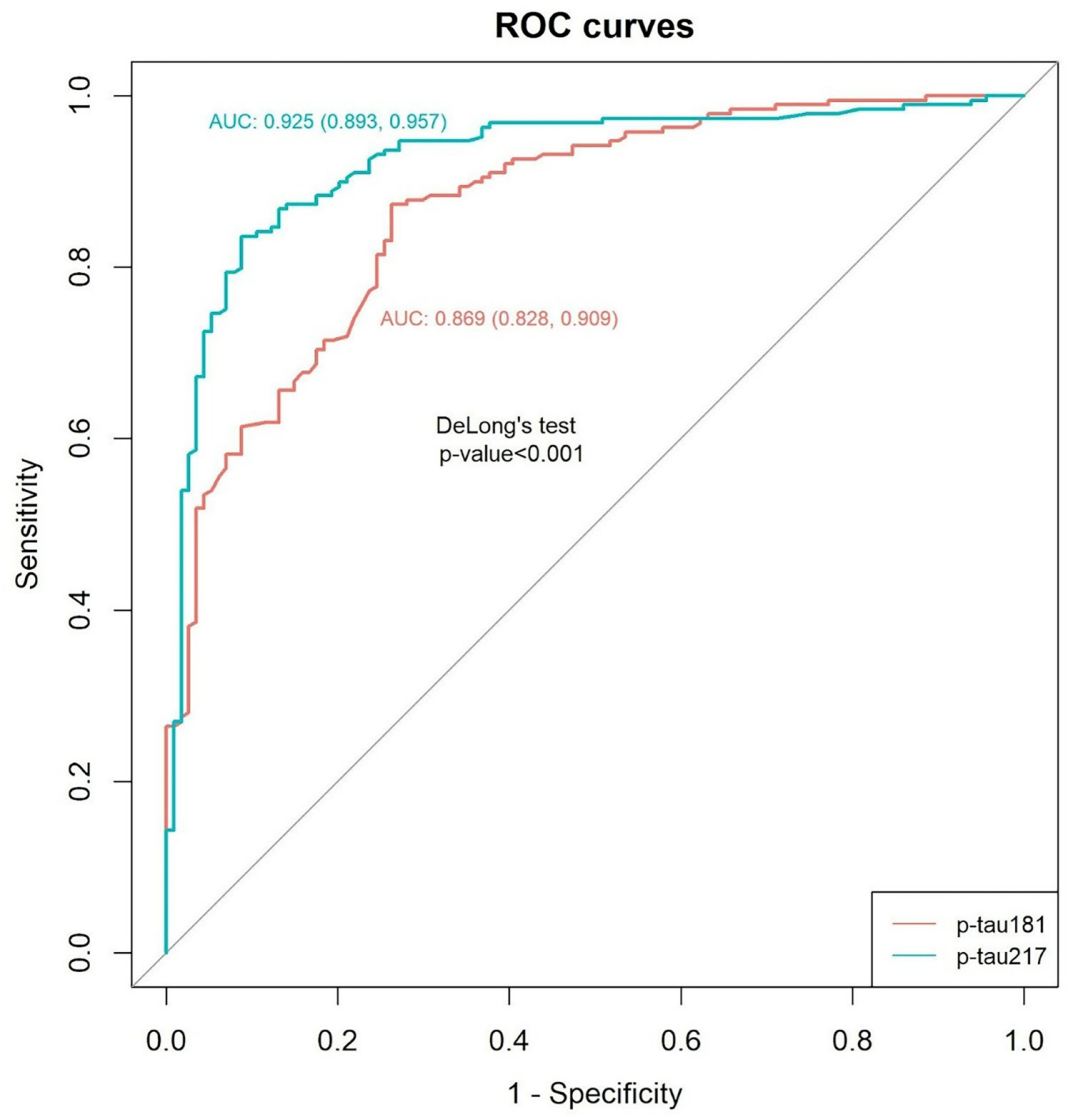
ROC curves comparing plasma p-tau181 versus plasma p-tau217 diagnostic utility.

For discrimination between AD and non-AD groups, plasma p-tau217 demonstrated superior diagnostic performance compared with p-tau181 (AUC = 0.93; 95% CI: 0.89–0.96 versus AUC = 0.87; 95% CI: 0.83–0.91), with the difference reaching statistical significance according to DeLong's test (*p* < 0.001) (Figure 2). When stratified by clinical stage, p-tau217 consistently outperformed p-tau181. In individuals with MCI, p-tau217 achieved excellent discrimination (AUC = 0.94; 95% CI: 0.90–0.97), markedly higher than that of p-tau181 (AUC = 0.86; 95% CI: 0.81–0.91) (*p* < 0.001). In patients with mild dementia, the performance gap between biomarkers was less pronounced, with p-tau217 yielding an AUC of 0.91 (95% CI: 0.84–0.98) compared with 0.89 (95% CI: 0.82–0.96) for p-tau181 (*p* = 0.365).

**Figure 3.**
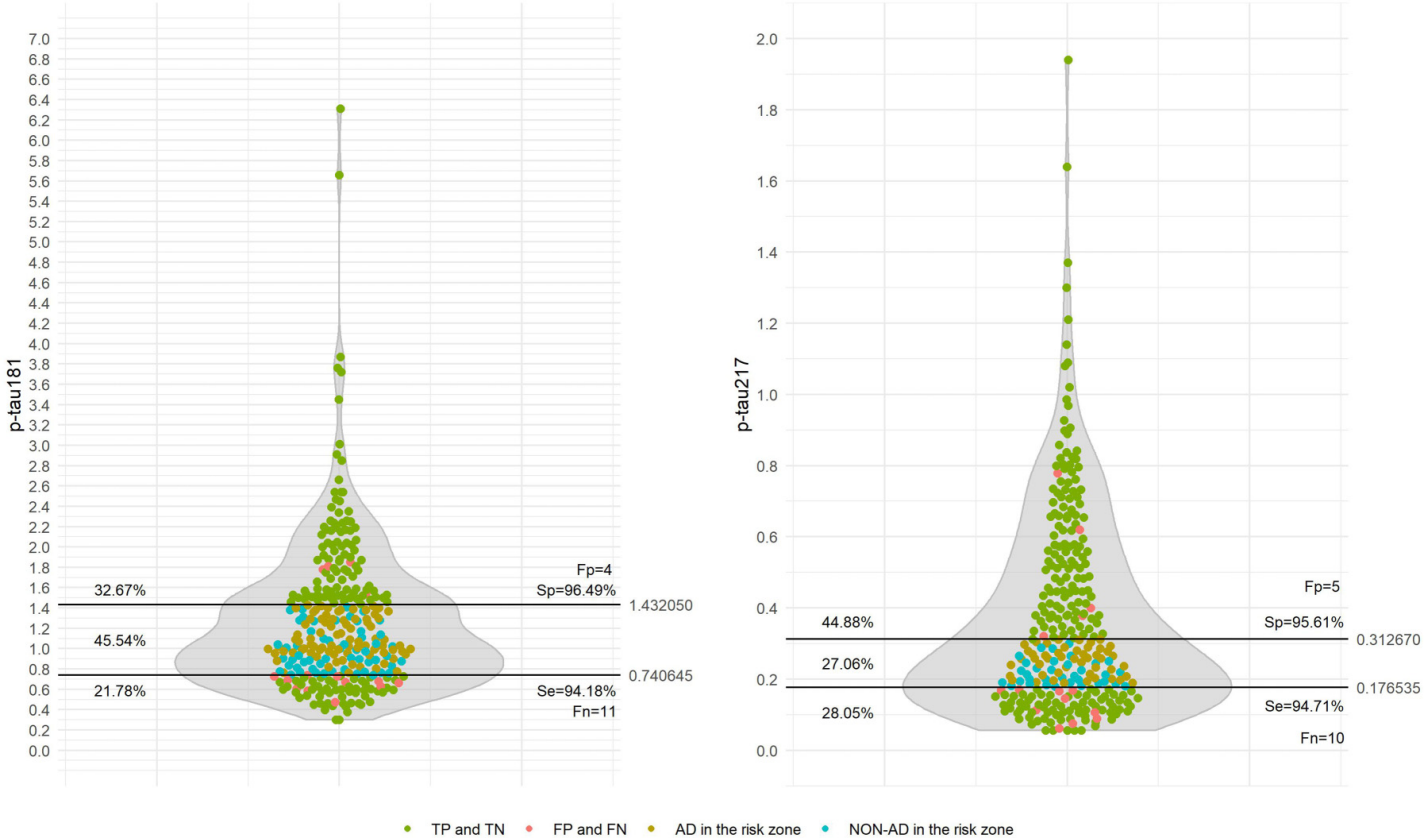
Plasma p-tau181 and p-tau217: distributions and cutoffs by 95% of sensitivity and specificity. Left: p-tau181 distribution and cutoffs; Right: p-tau217 distribution and cutoffs.

Using a two threshold strategy—prioritizing a specificity of ≥95% and the highest positive predictive value (PPV) for the upper cutoff, and a sensitivity of ≥95% and the highest negative predictive value (NPV) for the lower cutoff—thresholds of >1.432 pg/mL (PPV 96%) and <0.741 pg/mL (NPV 83%) were established for plasma p-tau181, leaving 46% of cases within the indeterminate (gray) zone (Figure 3A). Application of the same criteria to plasma p-tau217 resulted in cutoffs of >0.312 pg/mL (PPV, 96%) and <0.177 pg/mL (NPV, 88%), which reduced the proportion of gray-zone results to 27% (Figure 3B). Focusing on p-tau217, the overall accuracy reached 93%, outperforming p-tau181 (91%). Concordance analysis further supported the superior diagnostic performance of p-tau217, with 131 true positives and 75 true negatives, compared with 95 true positives and 55 true negatives for p-tau181. There were 5 false positives and 10 false negatives for p-tau217, and 4 false positives and 11 false negatives for p-tau181. Conducting a more detailed analysis, up to 40% of p-tau217 false positives could be suspected based on p-tau181 values below its upper cutoff (>1.432 pg/mL), and up to 70% of p-tau217 false negatives could be detected by p-tau181 values above its lower cutoff (<0.741 pg/mL).

The influence of glomerular filtration rate (GFR) on plasma p-tau217 and p-tau181 levels was evaluated in a subset of 188 patients who had GFR measurements available within one month of plasma biomarker assessment. Both plasma biomarkers showed statistically significant negative correlations with GFR (p-tau217: *r* = −0.279, *p* < 0.001; p-tau181: *r* = −0.375, *p* < 0.001). However, these associations were substantially weaker than the positive correlations observed between plasma p-tau biomarkers and the CSF p-tau181/Aβ42 ratio (p-tau217: *r* = 0.791, *p* < 0.001; p-tau181: *r* = 0.767, *p* < 0.001).

For both biomarkers, age showed an effect on diagnostic accuracy. When the cohort was stratified by the median age of 73 years, the area under the curve (AUC) for p-tau217 decreased from 0.94 (95% CI: 0.90–0.98) in younger individuals to 0.89 (95% CI: 0.82–0.96) in older individuals. A similar, though less pronounced, decline was observed for p-tau181, with AUCs decreasing from 0.88 (95% CI: 0.83–0.93) to 0.85 (95% CI: 0.77–0.92). Both differences were not significant. Focusing on plasma p-tau217 levels, a significant but weak positive correlation with age was detected in the complete cohort (*r* = 0.147; *p* = 0.011) and in the non-AD group (*r* = 0.217; *p* = 0.020). Accordingly, non-AD participants older than 73 years exhibited higher plasma p-tau217 concentrations than those aged 73 years or younger [0.18 (0.13–0.22) versus 0.14 (0.11–0.18); *p* = 0.019]. This age-related association was not observed in the AD group (*r* = -0.099; *p* = 0.174).

As expected, for the diagnosis of AD, other BBMs showed lower diagnostic accuracy than plasma p-tau217 and plasma p-tau181. The AUC for plasma GFAP was 0.82 (95% CI: 0.76–0.87), and for plasma NfL it was 0.64 (95% CI: 0.57–0.70). Several multivariable logistic regression models were constructed to assess whether the predictive performance of plasma p-tau217 could be improved by incorporating other variables that differed between AD and non-AD groups and to adjust for potential confounding variables (Table 3). Plasma GFAP and plasma NfL were excluded due to collinearity with plasma p-tau217 and p-tau181, and p-tau217 and p-tau181 were not included together in the same model (Table 3). The model with the highest AUC combined plasma p-tau217 with age, sex, and the APOE ε4p binarized result; however, this increase in AUC was not statistically significant, confirming the strong predictive ability of plasma p-tau217 alone (Table 3). GFR was not included in these models due to missing data in 115 patients. Nevertheless, a sensitivity analysis was performed. In the subset with available GFR data, plasma p-tau217 showed an AUC of 0.942 (95% CI: 0.908–0.977). Including GFR did not result in a statistically significant improvement (AUC = 0.944; 95% CI: 0.909–0.978; p = 0.41). As patients were included from two different cohorts, an additional sensitivity analysis incorporating cohort as variable was conducted. Plasma p-tau217 showed an AUC of 0.938 (95% CI: 0.897–0.979) in Cohort 1 (S2300168; ANRAC) and 0.924 (95% CI: 0.881–0.967) in Cohort 2 (S2300347), with no statistically significant differences between them (*p* = 0.654). Including cohort as a variable in the predictive models (with and without GFR) did not meaningfully change the results.

**Table 3.** Multivariable logistic regression models: diagnostic utility comparisons.

Model 1	AUC	Model 2	AUC	p
Plasma p-tau217	0.93	Plasma p-tau181	0.87	0.0036
Plasma p-tau217	0.93	Age + Sex + APOE ε4p	0.80	0.0001
Plasma p-tau217	0.93	Age + Sex + APOE ε4p + plasma p-tau217	0.94	1.0000
Plasma p-tau217	0.93	Age + Sex + APOE ε4p + plasma p-tau181	0.90	1.0000
Plasma p-tau181	0.87	Age + Sex + APOE ε4p	0.80	0.2003
Plasma p-tau181	0.87	Age + Sex + APOE ε4p + plasma p-tau217	0.94	0.0001
Plasma p-tau181	0.87	Age + Sex + APOE ε4p + plasma p-tau181	0.90	0.0780
Age + Sex + APOE ε4p	0.80	Age + Sex + APOE ε4p + plasma p-tau217	0.94	<0.0001
Age + Sex + APOE ε4p	0.80	Age + Sex + APOE ε4p + plasma p-tau181	0.90	<0.0001
Age + Sex + APOE ε4p + plasma p-tau217	0.94	Age + Sex + APOE ε4p + plasma p-tau181	0.90	0.0139

AUC: area under the curve. APOE ε4p (a concentration ≥ 0.9 µg/ml indicates *APOE* ε4 carrier status).

Plasma p-tau217 showed strong correlations with the CSF Aβ_42_/Aβ_40_ ratio (*r* = −0.766; *p* < 0.001) and the CSF p-tau181/Aβ_42_ ratio (*r* = 0.784; *p* < 0.001), as well as with plasma GFAP (*r* = 0.692; *p* < 0.001). Moderate correlations were observed with CSF t-tau (*r* = 0.496; *p* < 0.001), CSF p-tau181 (*r* = 0.596; *p* < 0.001), and plasma NfL (*r* = 0.443; *p* < 0.001).

**Figure 4.**
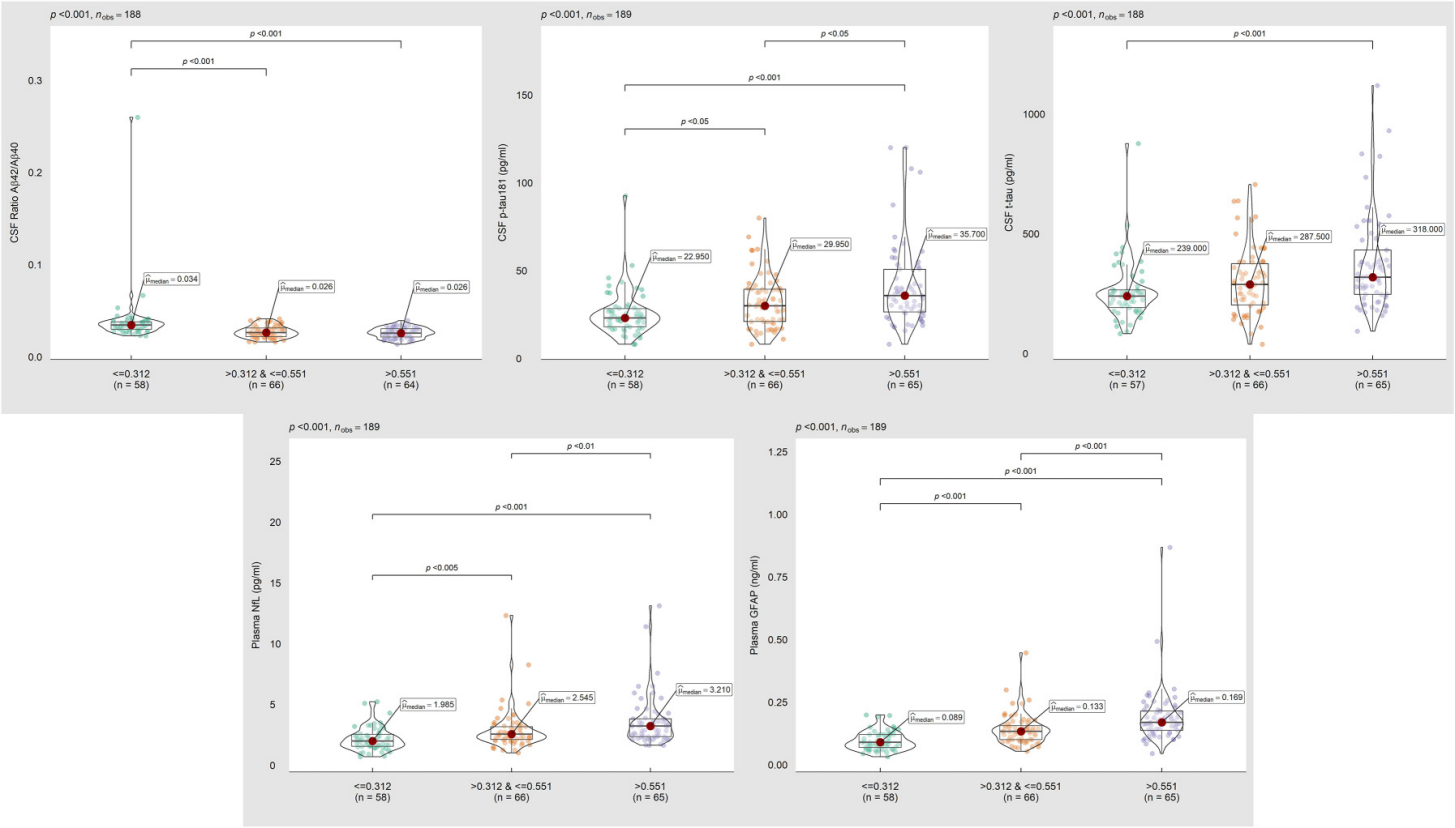
Comparison of CSF Aβ_42_/Aβ_40_ ratio, p-tau181 and t-tau, and plasma NfL and GFAP across plasma p-tau217 strata. (A) Intermediate and high plasma p-tau217 strata showed a significantly lower CSF Aβ_42_/Aβ_40_ ratio compared with the low plasma p-tau217 stratum. (B) CSF p-tau181 levels increased significantly across higher plasma p-tau217 strata. (C) CSF t-tau levels were significantly higher in the high plasma p-tau217 stratum compared with the low stratum. (D) Plasma NfL levels increased significantly across higher plasma p-tau217 strata. (E) Plasma GFAP levels increased significantly across higher plasma p-tau217 strata.

Table 4 presents the stratification of plasma p-tau217 values in AD patients into three strata. The first stratum (≤0.312 pg/mL) included patients diagnosed with AD whose p-tau217 results fell within the gray zone or were false negatives. The remaining two strata were defined by values below and above a new cutoff of 0.551 pg/mL, corresponding to the median of values above 0.312 pg/mL. These strata were associated with a stepwise increase in other soluble biomarkers, including markers of Aβ proteinopathy (Core 1: CSF Aβ_42_/Aβ_40_ ratio) and early tau pathology (Core 1: CSF p-tau181), as well as markers of neurodegeneration (CSF t-tau, plasma NfL) and neuroinflammation (plasma GFAP), with higher plasma P-tau217 levels corresponding to more pronounced changes in these biomarkers (Figure 4A-E).

**Table 4.** Biomarkers biological stratification by plasma p-tau217 intervals in patients with Alzheimer's disease.

Plasma p-tau217 level (pg/ml) n (%)	CSF ratio Aβ_42_/Aβ_40_	CSF t-tau (pg/ml)	CSF p-tau181 (pg/ml)	Plasma NfL (pg/ml)	Plasma GFAP (ng/ml)
≤0.312 pg/ml (n = 58, 30.7%)	0.034 (0.029-0.038)	239 (188–266)	23 (18–29)	1.98 (1.54–2.57)	0.09 (0.07–0.12)
>0.312-≤0.551 pg/ml (n = 66, 34.9%)	0.026 (0.021–0.031)	288 (197–380)	30 (21–39)	2.54 (2.09–3.18)	0.13 (0.10–0.16)
>0.551 pg/ml (n = 65, 34.4%)	0.026 (0.021–0.030)	318 (241–452)	36 (26–51)	3.21 (2.34–3.83)	0.17 (0.13–0.22)

Continuous variables are shown as median (interquartile range). AD: Alzheimer's disease; Non-AD: non-Alzheimer's disease; M: median; IQR: interquartile range; CSF: cerebrospinal fluid; NfL: neurofilament light chain; GFAP: glial fibrillary acidic protein.

## Discussion

This validation study reinforces the utility of plasma p-tau217 as a BBM for AD. Plasma p-tau217 demonstrated optimal diagnostic performance for identifying AD patients in a cohort where 70% of participants were at the MCI stage. In patients with AD, p-tau217 showed association with other soluble core 1, neurodegeneration, and neuroinflammation biomarkers.

The study was conducted on two cohorts of patients from specialized memory clinics. Among them, 62.3% were diagnosed with AD, while the remaining 37.6% comprised a heterogeneous group in which AD was excluded. In this group, confirmation of AD negativity, together with longitudinal clinical progression, has ultimately enabled the establishment of individualized diagnoses, including neurodegenerative conditions such as limbic age-related TDP-43 encephalopathy neuropathologic change, Lewy body dementia, and frontotemporal dementia with atypical memory presentation, as well as non-neurodegenerative causes such as cerebrovascular disease, atypical chronic adult hydrocephalus, and functional cognitive disorder. A remaining subset of cases with uncertain diagnoses still require clinical follow-up. These diagnoses reflect the diversity of specialized memory clinics, the setting of this validation study, where these plasma biomarkers will be implemented.

The diagnostic accuracy of plasma p-tau217 for distinguishing AD from non-AD patients exceeded that of p-tau181, confirming results from previous studies using Elecsys (“cobas”) and other platforms.^10,11,30^ While the diagnostic performance of p-tau217 was optimal, that of p-tau181 was only good. For p-tau217, an area under the curve (AUC) of 0.93 supports the implementation of the two threshold strategy.^10,11^ Compared with other validation studies using CSF as the reference standard and the two-threshold approach with 95% sensitivity and specificity, the resulting gray-zone proportion of 27% in our study is intermediate between the 19% and 32% previously reported.^10,31^ Notably, 58.5% of gray-zone samples were AD-positive. Although that 27% gray-zone exceeds the ≤20% often considered optimal to minimize the number of patients requiring additional testing (e.g., lumbar puncture or amyloid PET),^32,33^ this outcome in our cohort may be attributable to several factors when compared with other validation studies: a higher age in the non-Alzheimer's group, and the no exclusion of patients with CSF p-tau181/Aβ_42_ ratios near the 0.024 cutoff used as the diagnostic reference standard.

Using this two threshold strategy, the global accuracy agreement for plasma p-tau217 reached 93%, consistent with results reported for Lumipulse G measurements in secondary care settings and fulfilling the Alzheimer's Association's appropriate use recommendations for BBMs.^18,31,32,34^ The combination of a relatively high positive predictive value (96%) and a somewhat lower negative predictive value (88%) likely reflects the relatively high prevalence of AD (62%) in the study sample. With the selected p-tau217 cutoff points (upper: >0.312 pg/mL; lower: <0.177 pg/mL), 5 false positives and up to 10 false negatives were observed. These false negatives have also been reported in other p-tau217 and p-tau181 validation studies using a CSF reference standard, possibly because p-tau217 changes may not yet have occurred in some patients despite CSF positivity.^30^ These findings indicate that, although p-tau217 is a robust biomarker, it is not infallible, and particularly low values near the lower cutoff may benefit from complementary assessment with a second biomarker. For the Lumipulse platform, the plasma p-tau217/Aβ42 ratio has been proposed to clarify uncertain cases.^3,30^ In the present study, combined analysis of sensitivity and specificity for p-tau217 and p-tau181 suggested that up to 40% of p-tau217 false positives could be suspected based on p-tau181 values below its upper cutoff (>1.432 pg/mL), and up to 70% of p-tau217 false negatives could be detected by p-tau181 values above its lower cutoff (<0.741 pg/mL). In other words, despite the overall superior accuracy of p-tau217 compared with p-tau181, discordant p-tau181 results may, in specific cases, indicate false-positive or false-negative outcomes. This differing false-negative profile among p-tau epitopes warrants further investigation. On the cobas platform, until p-tau217 cutoff values are refined, combined assessment with p-tau181 may enhance diagnostic confidence.

Both plasma biomarkers, p-tau217 and p-tau181, were largely unaffected by variations in GFR, showing only weak associations compared with their correlations with the CSF p-tau181/Aβ_42_ ratio. This is consistent with previous validation studies reporting that GFR values below 45 mL/min are required to significantly affect these biomarker levels.^10,35^ Age, however, influenced the diagnostic performance of both biomarkers, with lower accuracy observed in older individuals. For p-tau217, this effect has been attributed to lower concentrations in older AD patients combined with higher concentrations in older participants without AD pathology.^13^ In our cohort, we confirmed a significant, weak, and positive correlation between age and p-tau217 levels in the non-AD group only, which may partially explain the reduced diagnostic utility of p-tau217 in older individuals. In this sense, some groups have highlighted that p-tau217 partly could reflect non-AD processes, including ageing, and the importance of tailored cut-off thresholds for cognitively unimpaired individuals aged ≥65 years, particularly if they are *APOE* ε4 carriers.^36,37^ Although the biomarker remains analytically robust, its specificity may decline with age, challenging a binary interpretation and increasing the relevance of clinical context and absolute plasma p-tau217 concentration when distinguishing AD from non-AD conditions.^11^ Additional positivity of core 2 AD biomarkers, as tau PET or, with growing evidence, MTBR-tau243, helps mitigate this limitation by identifying Aβ-positive individuals whose cognitive impairment is likely due to Alzheimer's disease pathology.^22^

Focusing on plasma p-tau217, its levels were strongly associated with the CSF Aβ_42_/Aβ_40_ ratio (reflecting amyloid burden), CSF p-tau181 (early tau proteinopathy), and plasma GFAP (neuroinflammation), and moderately associated with CSF t-tau and plasma NfL (markers of neurodegeneration).

In our study, the strong association between p-tau217 and other soluble CSF AD core 1 biomarkers confirms its role as a plasma AD core 1 biomarker with the ability to serve as a surrogate for CSF measures.^18^ Stratifying the AD group into three plasma p-tau217 strata revealed an associate stepwise increase on soluble Core 1, neurodegeneration, and neuroinflammation biomarkers. These strata reflect levels of amyloid pathology and may be useful for monitoring the response of soluble Core 1 biomarkers to anti-amyloid treatments, a response that has been demonstrated in clinical trials of lecanemab and donanemab.^2,38^ Empirically, the first stratum included the lowest p-tau217 values—those equal or below the upper cutoff of 0.312 pg/mL, corresponding to a specificity of 95%. This interval captures AD patients who fall within the gray zone or who are false negatives (below the lower cutoff of 0.177 pg/mL). The median p-tau217 value equal or above 0.312 pg/mL was 0.551 pg/mL, which was used to separate the intermediate and upper strata.

The study is limited to a panel of soluble AD core 1, neuroinflammation and neurodegeneration biomarkers available for Elecsys Automated Immunoassay. Observational and longitudinal studies evaluating plasma biomarkers, including p-tau217, GFAP, and NfL, and their changes in relation to amyloid and tau PET imaging have shown that p-tau217, as core 1 biomarker, progressively increases with rising Aβ burden and exhibits a rapid-then-slow upward trajectory during tau pathology accumulation.^39^

Plasma GFAP overexpression is recognized as a sensitive marker of astrocyte activation.^40^ Significant increases in brain tau pathology are observed in individuals presenting both Aβ deposition and abnormal GFAP levels. We observe a strong association in plasma between p-tau217 and GFAP. The cross-sectional design of the study and the lack of Core 2 biomarkers limit our ability to determine whether GFAP increases in response to amyloid burden or whether the observed association is mediated by tau pathology, reflecting more advanced AD pathology. Although GFAP is also considered a biomarker of non-specific processes involved in AD pathophysiology,^18^ in this study, based on its diagnostic performance and its correlations with plasma p-tau217, GFAP appears to be a more useful biomarker in AD than NfL. Supporting this assertion, GFAP has shown a greater response than NfL to anti-amyloid treatments in AD trials.^2,38^

Although higher plasma NfL levels in AD patients have also been associated with prospective cognitive decline,^41^ and faster longitudinal increases correlate with accelerated brain atrophy and hypometabolism, suggesting that NfL can serve as a noninvasive marker of neurodegeneration,^42^ the global role of plasma NfL in AD is less clear; it appears to be a less specific biomarker of the AD continuum than GFAP, showing only weak correlations with core AD pathologies and modest early increases during amyloid and tau accumulation on PET.^39^ In our study, although NfL also showed a stepwise increase according to plasma p-tau217 strata, this association was weaker compared with those observed for other soluble Core 1 biomarkers and GFAP, in line with its role as a more non-specific biomarker and its lower sensitivity to anti-amyloid therapies.^2,38^

One strength of our study is that it included only patients with MCI or mild dementia, representing the population in which assessment of amyloid pathology is most relevant for current amyloid-modifying therapies. Another strength is that these patients were recruited from a real-world clinical setting, making the cohort highly representative of routine clinical practice. The prospective inclusion of patients in both cohorts further strengthens the validity of this study. Lastly, the decision not to exclude patients with CSF p-tau181/Aβ_42_ ratios close to the diagnostic cutoff used as the reference standard emphasizes the discriminative capacity of plasma p-tau217 under routine clinical conditions.

Several limitations of this study should be acknowledged. First, it was conducted within a memory clinic setting; consequently, the findings may not be fully generalizable to the broader population or to other clinical contexts. Importantly, the reported negative and positive predictive accuracy of the two threshold strategy for plasma p-tau217 may vary depending on disease prevalence by different settings. In our memory clinic setting, the high prevalence of AD results in higher positive predictive value and lower negative predictive value. Second, the study population was relatively homogeneous and drawn from Andalusia; therefore, larger studies in more heterogeneous populations are needed to confirm these results. Third, an additional limitation is the lack of amyloid and tau PET imaging or neuropathological confirmation in our patients. Instead, the CSF p-tau181/Aβ_42_ ratio was used as the reference standard, and it remains unclear how the use of an alternative gold standard might have affected the results. The absence of Core 2 biomarkers, particularly tau-PET, limits the biological stratification of disease severity. Fourth, we used the plasma biomarker panel to measure *APOE* ε4p, which accurately separates *APOE* ε4 carriers from non-carriers.^29^ While this approach simplifies test logistics and implementation, it does not distinguish between one and two ε4 alleles, which is a disadvantage relative to traditional *APOE* genotyping.^29^ Finally, the cross-sectional design of the study, which primarily focused on BBMs validation, represents an additional limitation.

In conclusion, measured using the automated cobas platform, the Elecsys plasma p-tau217 demonstrated higher diagnostic utility for distinguishing AD from non-AD patients compared with p-tau181. While overall diagnostic performance was strong, the two threshold strategy was necessary. At 95% sensitivity and specificity, plasma p-tau217 achieved a global accuracy of 93% using the CSF p-tau181/Aβ_42_ ratio as the reference standard. Plasma p-tau217 also showed significant correlations with other soluble core 1 and neuroinflammation biomarkers.
